# The Role of Plasticity and Adaptation in the Incipient Speciation of a Fire Salamander Population

**DOI:** 10.3390/genes10110875

**Published:** 2019-10-31

**Authors:** Joana Sabino-Pinto, Daniel J. Goedbloed, Eugenia Sanchez, Till Czypionka, Arne W. Nolte, Sebastian Steinfartz

**Affiliations:** 1 Department of Evolutionary Biology, Zoological Institute, Technische Universität Braunschweig, 38106 Braunschweig, Germany; danielgoedbloed@hotmail.com (D.J.G.); eu.sanisa@gmail.com (E.S.); 2 Department of Biology, Stanford University, Stanford, CA 94305, USA; 3 Laboratory of Aquatic Ecology and Evolutionary Biology, KU Leuven, 3000 Leuven, Belgium; czypionka@evolbio.mpg.de; 4 Department of Ecological Genomics, Institute for Biology and Environmental Sciences, University of Oldenburg, 26129 Oldenburg, Germany; arne.nolte@uni-oldenburg.de; 5 University of Leipzig, Institute of Biology, Molecular Evolution and Systematics of Animals, 04103 Leipzig, Germany

**Keywords:** acclimatization, transplant experiment, phenotypic plasticity, local adaptation, transcriptomics

## Abstract

Phenotypic plasticity and local adaptation via genetic change are two major mechanisms of response to dynamic environmental conditions. These mechanisms are not mutually exclusive, since genetic change can establish similar phenotypes to plasticity. This connection between both mechanisms raises the question of how much of the variation observed between species or populations is plastic and how much of it is genetic. In this study, we used a structured population of fire salamanders (*Salamandra salamandra*), in which two subpopulations differ in terms of physiology, genetics, mate-, and habitat preferences. Our goal was to identify candidate genes for differential habitat adaptation in this system, and to explore the degree of plasticity compared to local adaptation. We therefore performed a reciprocal transfer experiment of stream- and pond-originated salamander larvae and analyzed changes in morphology and transcriptomic profile (using species-specific microarrays). We observed that stream- and pond-originated individuals diverge in morphology and gene expression. For instance, pond-originated larvae have larger gills, likely to cope with oxygen-poor ponds. When transferred to streams, pond-originated larvae showed a high degree of plasticity, resembling the morphology and gene expression of stream-originated larvae (reversion); however the same was not found for stream-originated larvae when transferred to ponds, where the expression of genes related to reduction-oxidation processes was increased, possibly to cope with environmental stress. The lack of symmetrical responses between transplanted animals highlights the fact that the adaptations are not fully plastic and that some level of local adaptation has already occurred in this population. This study illuminates the process by which phenotypic plasticity allows local adaptation to new environments and its potential role in the pathway of incipient speciation.

## 1. Introduction

Phenotypic plasticity and local adaptation are two major mechanisms of response to dynamic environmental conditions. These mechanisms appear under different population parameters and selection regimes. In changing environments, phenotypic plasticity allows organisms to conserve their genotypes and still produce alternative phenotypes [1,2,3,4], in contrast to local adaptations that incorporate changes at the genetic level producing long-lasting phenotypes and resulting in higher fitness in home habitats [5,6]. In theory, locally adapted traits are established via strong natural selection—even under conditions of high gene flow between locally and non-locally adapted populations [5,7]. In this situation, natural selection should also favour prezygotic isolation mechanisms to prevent mating between the populations [8,9,10], in doing so, this process can result in adaptive speciation (sensu Dieckman et al. [11]), which has been demonstrated for various groups of organisms [12,13,14].

Traditionally, phenotypic plasticity has been considered a force counteracting speciation because it buffers the effect of natural selection on plastic traits. However, modern research demonstrates that—by reinforcing the survival of organisms under varying environmental conditions—phenotypic plasticity exposes other traits to natural selection that are related but not directly involved in a given plastic response (plastic acclimatization) [15,16,17,18]. This way, plastic acclimatization serves as a key initial step towards the evolutionary divergence of other traits and characters [19,20]. Furthermore, some plastic responses are heritable, and if subject to selection can evolve via genetic adaptation (i.e., genetic accommodation) [21,22,23].

Amphibians constitute particularly good models to study the role of plasticity in evolution [24,25,26,27,28,29,30]. They offer numerous examples of plasticity [31,32], and are known for their variability with regards to behavior, morphology, physiology, and life history. The aquatic larvae of amphibians are of particular interest because many of the easily measurable morphological traits (such as size, weight, and gill length) are plastic and related to metamorphosis and fitness. A well-established natural system for evolutionary-ecological research is the European fire salamander (*Salamandra salamandra*); larvae of this species are capable of living in a variety of environments (streams, ponds, and underground springs) by changing their behavior and growth rate to the environmental conditions [33,34,35,36]. In Central Europe, *S. salamandra* larvae typically develop in small primary streams until metamorphosis [37], but females of some populations are known to deposit their larva in ephemeral water bodies, such as small ponds or ditches, and sometimes even in underground springs [30,35,38]. Both habitat types are ecologically different in many biotic and abiotic aspects, including water dynamics, temperature regime, food availability, and intraspecific competition [30,38]; for instance, ponds tend to have lower oxygen content and higher temperatures than streams [38,39,40]. One area where this kind of differential habitat use has been intensively studied is the range of the Ville forest in the West of Germany, with a special focus on the Kottenforst near Bonn. The Kottenforst represents a continuous old broad-leaf forest with streams and rain-filled bomb craters, in which adult salamanders can move freely and females deposit their larvae into small water bodies (streams or ponds), in which the latter develop until metamorphosis is completed. The adaptation to different larval habitat conditions has promoted the adaptive divergence of the ancestral local population into two genetically differentiated subpopulations, thus potentially representing a case of early stage ecological speciation [41]. 

In order to better study acclimatization and adaptation, a *S. salamandra* specific microarray chip was designed to estimate gene expression in different ecological contexts [42]. A previous study integrating results from gene expression in Kottenforst larvae under common environmental conditions revealed that the differential gene expression observed is mainly driven by transcriptional plasticity rather than local adaptation [43]. However, this was a laboratory-based study that focused on the effect of temperature and did not inform about the degree of plasticity in nature; it remains necessary to determine whether the patterns observed explain the variance observed in nature, or if other ecological parameters besides thermal adaptation drive the observed patterns. 

In this study, we performed a reciprocal transfer experiment of larvae between stream and pond habitats in the Kottenforst to estimate individual performance in the contrasting environments. Reciprocal transfer experiments—also known as transplant experiments—are experiments in which individuals are translocated from their native or original habitat to a new or ecologically different habitat [44,45,46,47], which allows the testing of heritability and fixation of traits in the natural environment. The aim of our transplant experiment was to test how far larvae can compensate for ecological differences between the two habitats. The performance of larvae was monitored using morphological variables and gene expression. Our results identify candidate genes underlying differential habitat adaptation in the fire salamander system, and illuminate their degree of plasticity and local adaptation.

## 2. Methods

### 2.1. Experimental Design

**Figure 1:**
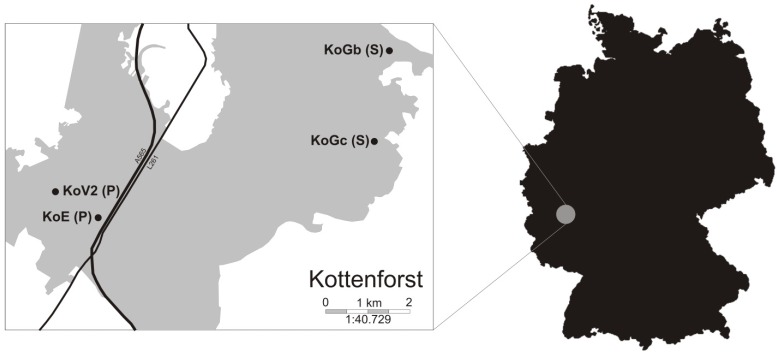
Map of the Kottenforst, Germany. The four salamander habitat sites included in the transfer experiment are indicated by black dots. Habitat type is indicated in brackets: P for temporary ponds, S for permanent streams. Black lines represent main roads (highway and state road).

**Figure 2:**
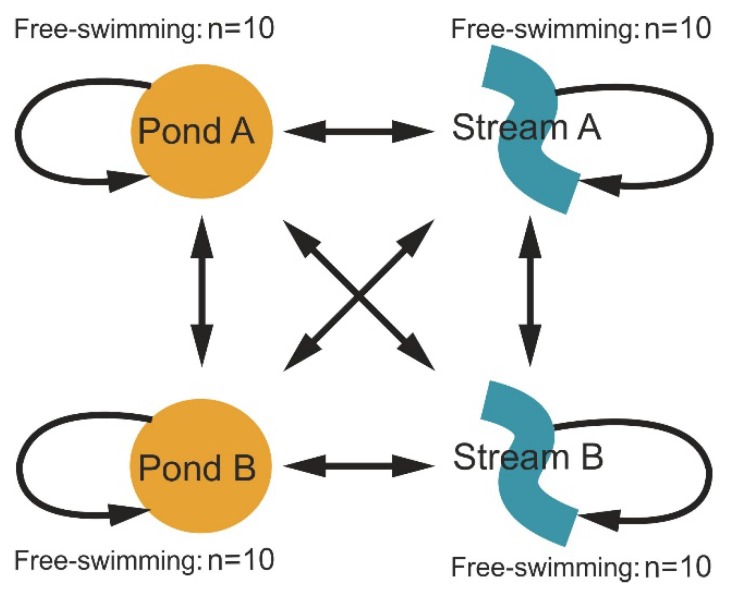
Experimental design. Representation of transfer design on which larvae from each site were transferred to each of the other sites in a fully reciprocal experiment. Each arrow represents 10 larvae. Additionally, 10 free swimming larvae were collected at each site.

Larvae of *S. salamandra* from both streams and ephemeral water bodies were captured with dipnets and used for a reciprocal transplant experiment at four sites in the Kottenforst (near Bonn, Germany). These sites included two permanent streams with flowing water (Klufterbach/KoGb and Vennerbach/KoGc) and two temporary ponds with stagnant water (KoE and KoV2) (Figure 1). We designed a full reciprocal transplant experiment in which 160 larvae—40 from each site—were transplanted to each of the four sites—10 were kept at the origin site, 10 were transferred to the other site of the same habitat type, 10 were transferred to one site of the other habitat type, and 10 were transferred to the other site of the other habitat type (Figure 2, Supplementary Table S1). Four treatment groups were set up: (1) S-S, stream-originated individuals kept in streams; (2) S-P, stream-originated individuals transferred to ponds; (3) P-S, pond-originated individuals transferred to streams; and (4) P-P, pond-originated individuals kept in ponds. A total of 160 larvae were individually housed in semi-permeable containers (1 L, HDPE plastic 8.65 × 8.65 × 17.85 cm with screw caps) which were equipped with two circular stainless-steel grid windows (5.0 cm diameter, mesh size 3.15 × 3.15 mm) and Styrofoam floaters (Supplementary Figure S1). The mesh size of the grid windows was chosen so that the smallest salamander larvae could not escape, while allowing potential food items to enter the container. Larvae were randomized when determining the destination site; containers were placed randomly at each site along the margins, far enough from the edge to ensure that the entire container was underwater but not buried. Larvae to be placed in the experimental containers were collected at different points of the streams/ponds to avoid maternal effects; additionally, newborns (with a ventral yolk patch) and larvae at prometamorphosis or metamorphic climax were excluded to avoid bias introduced through the inclusion of different developmental stages [48].

### 2.2. Sampling and Morphology Measurements

Larvae were captured and placed in the containers on 11 May 2015, and the experiment lasted 14 days. At the start of the experiment each larva was weighed and photographed from the top and side. Photographs were taken in a custom Plexiglas cuvette with a millimeter paper background for morphological measurements (Supplementary Figure S2). At the end of the experiment, larvae were weighed, photographed, and tail-clipped. Tail-clips were stored in RNA-later and kept at −20 °C, until further analysis. Tail-clipping is typically used when tissue-sampling salamander larvae due to their ability to regenerate their tail, and the demonstrated lack of adverse effect on general performance [49]. Furthermore, tail-clips provide high-quality data for gene expression analysis [50]. Additionally, 10 free roaming larvae were collected at each site, and their tails were clipped to serve as a morphology/gene expression control for potential container effects.

### 2.3. Morphological Data Analysis

As an experimental control (habitat differences), we first compared individuals that were not translocated between habitat types (P-P versus S-S). Then, to explore the individual adaptation (adaptive differences), we compared individuals translocated between different habitat types to individuals that were not translocated between habitat types (P-P versus P-S, P-S versus S-S, S-S versus S-P, S-P versus P-P) (Figure 2).

The snout-vent length (SVL) and the length of the longest rachis of the right gill on top and side pictures were measured with ImageJ software. Each measurement was done in triplicate and the average used for further analysis. All statistical analyses were performed in R [51]. Gill length (GL) was calculated as the average between lateral gill length and top gill length, and corrected for the SVL to allow for comparison. Body condition index (BC) was calculated as SVL divided by weight. Growth rate (hereafter referred to as Growth) was extracted from Bletz et al. [47] with the formula: G = (ln(Wt+1) − ln(Wt))/t; where, Wt is mean larval fresh weight at the start, Wt+1 is fresh weight at the end of the experiment (guts from all individuals were full at this point [47]), and t indicates the time period (days) between the start and end of the experiment. In order to determine if there were differences in BC, GL, and Growth between treatment groups, independent linear models (LM) were calculated with treatment as the factor. The normal distribution of the residuals was controlled with Shapiro-Wilk tests and the homogeneity of variance with Levene’s tests. The BC had to be log (x) transformed to allow for normal residual distribution. For the adaptive differences, groups were compared with a one-way ANOVA with subsequent Tukey *post hoc* tests (function ghlt, package multcomp [52]).

### 2.4. Gene Expression Analysis

For each of the surviving larvae (N = 93, Supplementary Table S2) the total RNA was extracted using a TRIzol protocol and hybridized to an Agilent microarray representing 12,744 *S. salamandra* genes known to be expressed at the larval stage [42]. The TRIzol protocol was based on that provided by ThermoFisher Scientific TRIzolTM Reagent (Thermo Fisher Scientific) with the following changes: (1) Step 2b: Centrifugation at 12,000 G for 15 min; (2) Step 2d: Drying for 1 min [42]. The extracted RNA was hybridized with the microarray and the fluorescent label signal intensities were quantified in an Agilent DNA Microarray Scanner type C. Signal intensities were normalized using a custom R script, which included a correction for probe-binding behavior [50], as well as the merging of technical replicates by taking their median and 75 percentile between-array normalization, following the recommendations in the Agilent ‘One-Color Microarray-Based gene expression Analysis’ protocol [42].

To explore patterns of gene expression, we used *a priori* hierarchical clustering based on a Pearson correlation matrix (function rcorr, package Hmisc [53]), and principal component analysis (PCA) (function PCA, package FactoMineR [54]; function fviz_pca_ind, package factoextra [55]). Differences between groups were tested with a PERMANOVA using the treatments as factors (function adonis, package vegan [56]). To identify transcripts that were significantly differentially expressed between larvae from the stream and pond habitat, the linear discriminant analysis effect size (LEfSe) method was employed [57], as implemented in the Galaxy platform (http://huttenhower.sph.harvard.edu/galaxy/), with treatment as class and habitat of origin as subclass, and using default parameters. To allow for a better visualization of the data, *a posteriori* PCAs were made using only the probes identified by the LEfSe analysis. Expression patterns of differently expressed transcripts were identified with self-organizing maps (SOM) learning machine (package oposSOM [58]). Transcript annotation—including gene ontology (GO) terms relationships—was performed as described in Sanchez et al. [48]. Grouping of biological process GO (BP-GO) terms was based on the parental-child relationships of these GO terms, and were visualized in the platform QuickGO (http://www.ebi.ac.uk/QuickGO). Additionally, GO terms were used for gene enrichment analysis (Fisher exact test, package topGO [59]), biological processes were weighted by their *p*-values with cutoff values of 0.05.

The results obtained from the comparison between P-P and S-S individuals in this study were compared to two other studies that focused on the same *S. salamandra* population from the Kottenforst [42,43]. These two studies were based on the same data set, with Czypionka and Goedbloed et al. [43] using a subset of the data from Goedbloed et al. [42] with additional transcriptome data from laboratory experiments. Since all three studies used the same microarray chip, the IDs of the probes associated with differently expressed transcripts could be directly compared.

Gene expression files are deposited at the GEO omnibus repository (GSE139590).

## 3. Results

### 3.1. Habitat Differences

**Figure 3:**
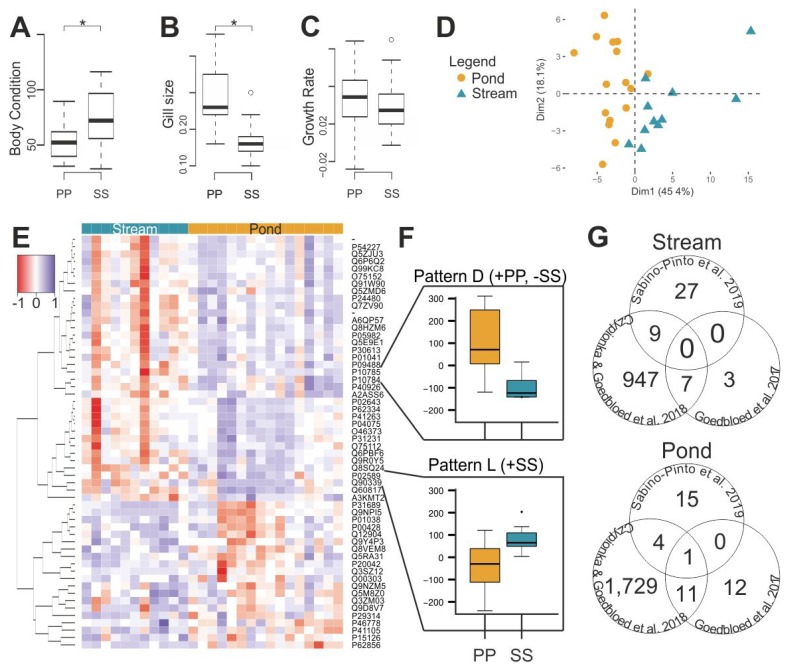
Habitat type experiment results. Histograms representing (**A**) body condition index, (**B**) gill size, and (**C**) growth rate of pond (PP) and stream (SS) individuals. (**D**) *A posteriori* principal component analysis of gene expression data depicting pond (circles) and stream individuals (triangles). (**E**) Heat map of the differently expressed transcripts (56) between PP and SS. (**F**) Expression patterns identified by self-organizing maps analysis based on differently expressed probes (+: Overexpression, -: Underexpression). (**G**) Venn diagrams showing overexpressed transcripts in streams and ponds shared between this study and the studies of Goedbloed et al. [42] and Czypionka and Goedbloed et al. [43].

P-P and S-S individuals differed from each other (Figure 3, Supplementary Figure S3A–C) in body condition, gill size, and gene expression. There was no effect related to translocation of individuals between sites of the same habitat type (e.g., PondA-PondA versus PondA-PondB) or cage-effect in our experiments (see Supplementary Results).

The body condition index (BC) of S-S individuals was significantly higher than those of P-P individuals (Stream: $\bar{x}$ = 73.56 ± 24.14; Pond: $\bar{x}$ = 52.76 ± 16.24; ANOVA: F_1,26_ = 17.70, *p*-value < 0.001; Figure 3A), while the gills (GL) were significantly larger in the P-P individuals (Stream: $\bar{x}$ = 0.135 ± 0.019; Pond: $\bar{x}$ = 0.193 ± 0.037; ANOVA: F_1,26_ = 73.86, *p*-value < 0.001; Figure 3B). During the period of the experiment, the growth rate (Growth) for both groups was similar (Stream: $\bar{x}$ = 0.014 ± 0.013; Pond: $\bar{x}$ = 0.009 ± 0.015; ANOVA: F_1,26_ = 2.44, *p*-value = 0.123; Figure 3C). 

Of the 12,744 probes present in the microarray, 12,419 had no missing values and were used in the analysis. An *a posteriori* PCA of the data revealed grouping of the samples according to habitat type (PERMANOVA: F_1,26_ = 0.12, *p*-value < 0.001; Figure 3D); which was not clear in the *a priori* display of the data (Supplementary Figure S3A,B). For the S-S versus P-P comparison, there were 56 (0.5%) probes targeting differently expressed transcripts: 36 (64.3%) were overexpressed in S-S and 20 (35.7%) in P-P individuals (Figure 3E). The identified probes clustered in 12 expression patterns, with pattern D (N = 2) being underexpressed in S-S and overexpressed in P-P; and pattern L (N = 2) being overexpressed in S-S individuals (Figure 3F, Supplementary Figure S3C). Of these 56 probes, 35 (62.5%) were associated with BP-GO terms: 24 (68.6%) were overexpressed in S-S, and 11 (31.4%) were overexpressed in P-P (Supplementary Table S3). The enrichment analysis of the 36 transcripts overexpressed in S-S individuals revealed nine specific overrepresented GO terms, which were associated with “response to vitamin A” and “DNA damage response, detection of DNA damage”. The enrichment analysis of the 20 transcripts overexpressed in P-P revealed seven specific overrepresented GO terms which were associated with “peptide cross-linking” and “positive regulation of neutrophil apoptotic process” (Supplementary Table S4).

When comparing the results obtained here to those of previous studies on *S. salamandra* [42,43], there was some overlap in the differentially expressed transcripts (Figure 3G). A total of 25% of the probes targeting transcripts overexpressed in S-S and P-P individuals (9/36 and 5/20, respectively), in this study were also overexpressed in the other two studies. The BP-GO terms overexpressed in S-S shared between the studies were related to “translation”; while those overexpressed in P-P were related to “DNA repair”, “phosphorelay signal transduction system”, and “DNA recombination” (Supplementary Table S5).

### 3.2. Evolutionary Divergence

**Figure 4:**
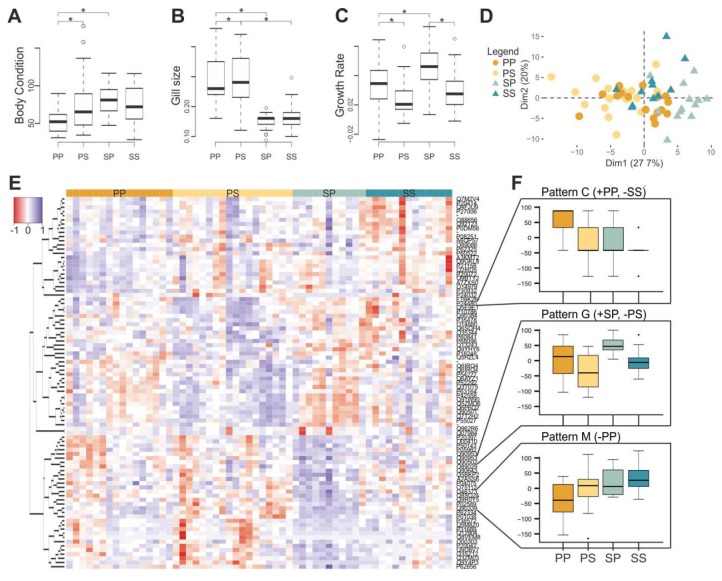
Transfer experiment results. Histograms representing (**A**) body condition index, (**B**) gill size, and (**C**) growth rate of pond individuals (PP), pond individuals transferred to streams (PS), stream individuals transferred to ponds (SP) and stream individuals (SS). (**D**) *A posteriori* principal component analysis of gene expression data depicting pond-originated (circles), stream-originated individuals (triangles), transferred (lighter), and non-transferred (darker). (**E**) Heat map of the differently expressed transcripts (89) between PP, PS, SP, and SS. (**F**) Expression patterns identified by self-organizing maps analysis based on differently expressed probes (+: Overexpression, -: Underexpression).

Individuals that were transferred between habitat types (Figure 2) presented divergent responses from those not transferred between habitat types, both in fitness (body condition, gill length change, and growth), and in gene expression (Figure 4, Supplementary Figure S3D–F). During the final days of the experiment, ambient temperatures drastically rose (>25 °C), which coincided with mortality of some of the larvae—particularly in the S-P group (Supplementary Table S2). 

The body condition index (BC) and the gill length (GL) of S-P individuals were equal to those of S-S individuals, and significantly different from those of P-P individuals (Tukey BC − SS-SP: *t* = −1.42, *p*-value = 0.492; PP-SP: *t* = 5.21, *p*-value < 0.001; Tukey GL − SS-SP: *t* = 1.35, *p*-value = 0.530; PP-SP: *t* = −9.23, *p*-value < 0.001). The growth rate (Growth) of S-P individuals was significantly different than that of both the S-S and P-P individuals (Tukey Growth − SS-SP: *t* = −5.35, *p*-value < 0.001; PP-SP: *t* = 9.93, *p*-value < 0.001; Figure 4A–C). The BC and Growth of P-S individuals did not differ from those of S-S individuals, but was significantly different from that of P-P individuals (Tukey BC − SS-PS: *t* = 0.33, *p*-value = 0.988; PP-PS: *t* = 3.74, *p*-value = 0.002; Tukey Growth − SS-PS: *t* = 1.57, *p*-value = 0.397; PP-PS: *t* = −3.24, *p*-value = 0.008); while the gill size was equal to that of P-P individuals, but significantly different from that of the S-S individuals (Tukey Gill − PP-PS: *t* = 0.33, *p*-value = 0.988; SS-PS: *t* = −8.63, *p*-value < 0.001; Figure 4A–C).

Adding the data of the additional transplant samples to the analysis reduced the number of transcripts with no missing values from 12,419 to 12,291. An *a posteriori* PCA of the data revealed grouping of the samples according to treatment (PERMANOVA: F_3,57_ = 6.49, *p*-value < 0.001; Pairwise PERMANOVA: PP-SP: F_1,57_ = 9.32, *p*-value = 0.006; SS-SP: F_1,57_ = 3.90, *p*-value = 0.006; PP-PS: F_1,57_ = 2.92, *p*-value = 0.120; SS-PS: F_1,57_ = 4.89, *p*-value = 0.006; Figure 4D); which was not clear in the *a priori* display of the data (PERMANOVA: F_3,57_ = 0.15, *p*-value < 0.001; Supplementary Figure S3D,E). There were 89 (0.7%) probes targeting differently expressed transcripts: 13 (14.6%) were overexpressed in P-P individuals, 26 (29.2%) in P-S, 29 (32.6%) in S-P, and 21 (23.6%) in S-S (Figure 4E). The identified probes clustered in 13 expression patterns, with pattern C (N = 1) being underexpressed in S-S individuals, and overexpressed in P-P; pattern G (N = 2) being underexpressed in P-S individuals, and overexpressed in S-P; and pattern M (N = 5) being underexpressed in P-P individuals (Figure 4F, Supplementary Figure S3F). Of the 89 probes, 49 (55.1%) were associated with BP-GO terms; six (12.2%) were overexpressed in P-P individuals, 11 (22.4%) in P-S, 17 (34.7%) in S-P, and 15 (30.6%) in S-S (Supplementary Table S6). The enrichment analysis of the 13 transcripts overexpressed in P-P individuals revealed three specific overrepresented GO terms that were associated with “cellular water homeostasis” and “cellular response to estradiol stimulus”. The enrichment analysis of the 26 transcripts overexpressed in P-S individuals revealed ten specific overrepresented GO terms, which were associated with “keratinization” and “membrane repolarization”. The enrichment analysis of the 29 transcripts overexpressed in S-P individuals revealed seven specific overrepresented GO terms, which were associated with “skeletal system development” and “mitotic chromosome condensation”. The enrichment analysis of the 21 transcripts overexpressed in S-S individuals revealed ten specific overrepresented GO terms, which were associated with “amino-acid betaine catabolic process” and “DNA damage response, detection of DNA damage” (Supplementary Table S7).

## 4. Discussion and Conclusions

Our results demonstrate that stream and pond salamanders differ in their regulatory networks, not only due to phenotypic plasticity, but also due to local adaption to their home habitats. The lack of symmetrical responses in the treatments where individuals were transferred between habitats shows that the divergence of stream- and pond-originated salamanders is at least partly genetically based and not fully plastic; this highlights the potential for adaptive evolution in this population. These findings are further reinforced by genetic data that supports the observation that stream- and pond-originated salamanders are two genetically differentiated subpopulations [41]. Moreover, this study provides novel data on the ability of individuals to adapt to the ancestral habitat type (regression), and the challenges of colonizing new habitats.

**Figure 5:**
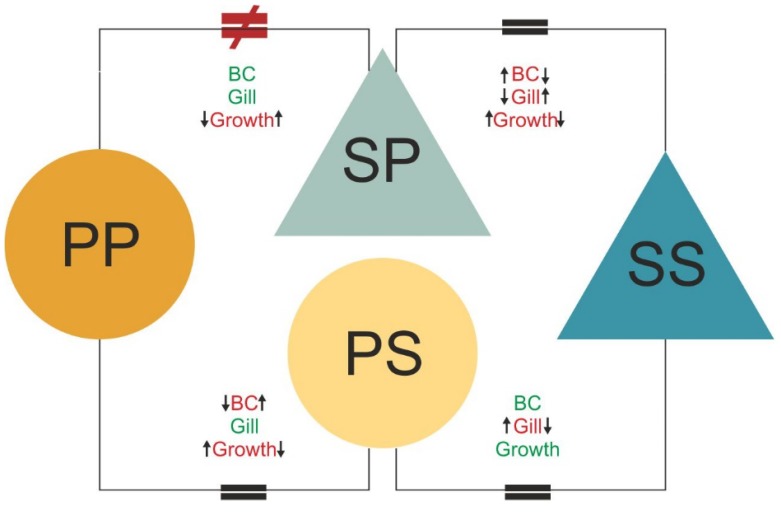
Schematic representation of physiological and transcriptomic results in response to habitat alteration. Circles and triangles represent treatment groups: P-P: Non-transferred pond individuals; P-S: Pond individuals transferred to streams; S-P: Stream individuals transferred to ponds; S-S: Non-transferred stream individuals. Text between treatment groups represents physiological results: BC: Body condition index; Gill: Gill length; Growth: Growth rate; and the arrows represent direction of the differences between groups. Connections between treatments represent similarity (=) and dissimilarity (≠) observed in transcriptomic responses.

From the morphological data, pond-originated larvae had lower body condition, but larger gills (Figure 3 and Figure 5). The lower body condition is likely related to the lower amount of food available in ponds [47], while the bigger gills could develop as a way to absorb more oxygen in a low-oxygen environment. This second aspect relies on the oxygen- and capacity-limited thermal tolerance (OCLTT) theory [60,61], which states that the performance of aquatic organisms varies at different environmental temperatures due to a mismatch between the metabolic demand for oxygen and the supply of oxygen to the tissues. Further studies should explore the specific role of oxygen in the performance of stream- and pond- originated larvae.

Gene expression differences between stream- and pond-originated larvae in the Kottenforst detected in previous studies [42,43] are here confirmed. Of the probes found to target differentially expressed genes in those studies, 25% were also identified as differentially expressed in this study. Although this overlap seems small when compared to the ~50% overlap between those studies (Figure 3G), we must emphasize that those studies were largely based on the same data whereas herein we analyze a new dataset.

In both our experiments, stream- and pond-originated larvae had different enriched GO terms derived from differentially expressed transcripts, further underscoring the variation in needs for each of the habitats. Lentic environments tend to be unstable, which has been linked to the observation that metamorphosis of pond-originated larvae occurs earlier [30]. We found that pond-originated larvae had enrichment in peptide cross-linking, which is important in generating mechanically stable structures such as skin and cartilage, and is known to increase in expression during maturation/metamorphosis [62]. These larvae also showed enrichment in positive regulation of neutrophil apoptosis, which is enhanced during metamorphosis to prevent auto immune responses [63]. On the other hand, solar radiation in streams is higher than in ponds, potentially explaining the enrichment in response to vitamin A seen in stream-originated larvae, as vitamin A is known to reduce radiation-induced difficulties in healing [64].

The reciprocal transfer experiment provides further evidence of the adaptive potential of stream- and pond-originated individuals to their environment, and therefore provides further evidence that the stream and pond-breeding subpopulations of *S. salamandra* in the Kottenforst are diverging from one another. We find that P-S individuals presented a gene expression profile that is intermediate between those of pond (origin) and stream (destination) larvae, showing some acclimatization to the stream environment (regression). This is further supported by the morphological data (Figure 4 and Figure 5)—the gill length of P-S individuals matched that of the pond individuals—yet the body condition index and the growth rate matched that of stream individuals(Figure 5); this is likely due to the abundance of food in this environment [47]. Conversely, S-P individuals presented a gene expression profile similar to individuals from streams (origin) but not to those from ponds (destination). The reduced acclimatization to the destination environment can also be seen in the morphological data, in which the body condition index and the gill length of S-P individuals is the same as that of S-S individuals (Figure 5). The only aspect of S-P individuals which did not match S-S individuals was the growth rate; this was higher than both non-transferred groups (P-P and S-S; Figure 4C and Figure 5). This could be related to the fact that streams have lower oxygen content and food availability [38,39,47]. These differences are potentially interpreted as environmental stress and induce hormonal changes [65,66,67] and, thus lead to more rapid metamorphosis [68,69]. This is further supported by the fact that S-P individuals had enriched GO terms related to skeletal system development and mitotic chromosome condensation, which are functions associated with growth and metamorphosis.

Streams are thought to be the ancestral breeding site of *S. salamandra*, because most populations deposit their larvae in streams [37], including the population of *S. salamandra* closest to the Kottenforst, in the Eifel, Germany [41]. This explains why pond-originated salamanders were able to acclimatize to streams (regression), while the stream-originated larvae—never having been in contact with the pond environment before—could not acclimatize as easily to the conditions imposed by ponds. Combining these results with the genetic structure of the Kottenforst *S. salamandra* population [41] led us to conclude that divergent selection is occurring in the ponds. This force probably led to local adaptations in the pond-originated larvae that has potentially been maintained through preferential mate choice [70]. 

The value of diversification over homogenization of the population can be linked with the exploitation of new resources to avoid intraspecific competition. This idea is further supported by differences in the gut microbiota between these subpopulations, which allow them to be more efficient at acquiring nutrients in their preferred habitat [47].

Together with other studies that show differences between both sub-populations of *S. salamandra* in the Kottenforst, related to physiological aspects [30], genetic clusters [41], mate preference [70], and gene expression [42,43], this study provides further evidence on how adaptation to new environments can lead to incipient divergence/speciation. These sub-populations seem to be accumulating differences at various levels that may cumulate in the formation of different subspecies. This early phase of speciation is often difficult to detect, and therefore this population of *S. salamandra* provides a rare and ideal study system to test microevolution questions regarding how speciation through phenotypic plasticity occurs.

## Supplementary Materials

The following are available online at https://www.mdpi.com/2073-4425/10/11/875/s1. Supplementary Results: Container effect, Figure S1: Field setup. (A) Placement of two sets of containers at KoV2. (B) Example container used to house the larvae during the experiment, Figure S2: Examples of top (A) and side (B) photos of the same salamander larva, Figure S3: Habitat type (A–C) and transfer (D,E) experiment results. (A) and (D) *a* *priori* principal component analysis, (B) and (E) hierarchical clustering based on a Pearson correlation distance matrix of gene expression data, (C) and (F) expression patterns identified by self-organizing maps analysis based on differently expressed probes without differences between groups. PP: Pond individuals; PS: Pond individuals transferred to streams; SP: Stream individuals transferred to ponds; SS: Stream individuals, Figure S4: Cage effect experiment results. (A) *A priori* principal component analysis, and (B) hierarchical clustering based on a Pearson correlation distance matrix of gene expression data depicting experimental individuals (triangles) and free-swimming ones (circles). P for pond individuals, S for stream individuals, Table S1: Experimental design. Number of individuals transferred between sites in a fully reciprocal experiment. In addition, 10 free swimming larvae were collected at each site, Table S2: Sample sizes. Groups are coded in a from-to fashion based on site ID (B=KoGb, C=KoGc, E=KoE, and V=KoV2), Table S3: List of probes differently expressed between larvae originated from ponds and streams in the Kottenforst. “LDA effect size” and “*p*-value” according to LEfSe, “Functions” according to QuickGO, transcript description according to Annocript, Table S4: GO term (code and name) enrichment for habitats. Site: Habitat on which terms were upregulated. Significant: p(FDR): Significance levels of GO terms that were enriched with pFDR < 0.05, Table S5: List of probes differently expressed between larvae originated from ponds and streams in the Kottenforst according to Czypionka et al. (2015), Goedbloed et al. (2016), and this study. “Function” according to QuickGO, transcript description according to Annocript, Table S5: List of probes differently expressed between larvae from the Kottenforst originating from ponds and kept in ponds (PP), originating from ponds and transferred to streams (PS), originating from streams and transferred to ponds (SP), and originating from streams and kept in streams (SS). “LDA effect size” and “*p*-value” according to LEfSe, “Functions” according to QuickGO, transcript description according to Annocript, Table S7: GO term (code and name) enrichment for treatments. Treatment: Treatment on which terms were upregulated. Significant: p(FDR): Significance levels of GO terms that were enriched with pFDR < 0.05, Table S8: Morphological raw data. (B = KoGb, C = KoGc, E = KoE, and V = KoV2).
