# Erratum: The Role of Plasticity and Adaptation in the Incipient Speciation of a Fire Salamander Population (*Genes* 2019, *10*, 875)

**DOI:** 10.3390/genes11040399

**Published:** 2020-04-07

**Authors:** Joana Sabino-Pinto, Daniel J. Goedbloed, Eugenia Sanchez, Till Czypionka, Arne W. Nolte, Sebastian Steinfartz

**Affiliations:** 1 Department of Evolutionary Biology, Zoological Institute, Technische Universität Braunschweig, 38106 Braunschweig, Germany; joanasabinopinto@gmail.com (J.S.-P.); danielgoedbloed@hotmail.com (D.J.G.); eu.sanisa@gmail.com (E.S.); 2 Department of Biology, Stanford University, Stanford, CA 94305, USA; 3 Laboratory of Aquatic Ecology and Evolutionary Biology, KU Leuven, 3000 Leuven, Belgium; czypionka@evolbio.mpg.de; 4 Department of Ecological Genomics, Institute for Biology and Environmental Sciences, University of Oldenburg, 26129 Oldenburg, Germany; arne.nolte@uni-oldenburg.de; 5 University of Leipzig, Institute of Biology, Molecular Evolution and Systematics of Animals, 04103 Leipzig, Germany

## 1. Error in Figure

In the original article, there was a mistake in Figure 5 as published. When summarizing the results in the scheme, the treatment groups were mixed, and so some of the symbols for morphological and gene expression traits were not in accordance with the results. The corrected Figure 5 appears below. The authors apologize for this error, and state that this does not change the general scientific conclusions of the article in any way (the variability in the study population is still partially due to plasticity and partially due to genetic adaptation, however the direction is reversed. Since the discussion and abstract were written with basis on the mistaken figure there are a couple of sentences that need correction: see below).

**Figure 5:**
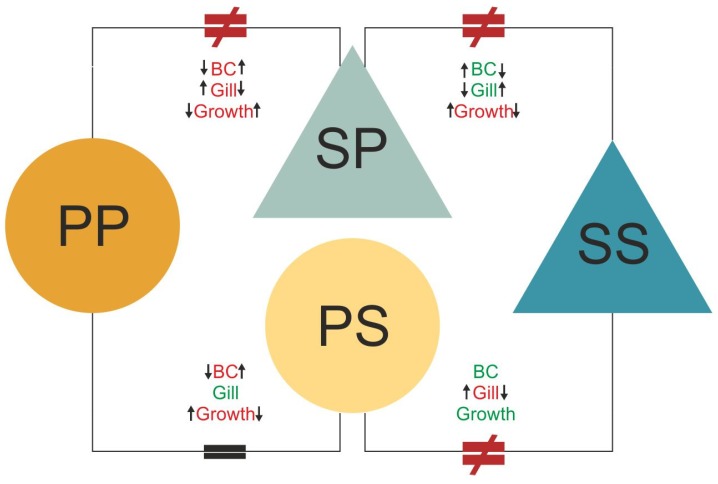
Schematic representation of physiological and transcriptomic results in response to habitat alteration. Circles and triangles represent treatment groups: P-P: Non-transferred pond individuals; P-S: Pond individuals transferred to streams; S-P: Stream individuals transferred to ponds; S-S: Non-transferred stream individuals. Text between treatment groups represents physiological results: BC: Body condition index; Gill: Gill length; Growth: Growth rate; and the arrows represent direction of the differences between groups. Connections between treatments represent similarity (=) and dissimilarity (≠) observed in transcriptomic responses.

## 2. Text Correction

In the original article, there was an error. Since the discussion and abstract were written with a basis on the mistaken figure, there are a couple of sentences that need correction. Most corrections refer to the treatment name—additional changes were done in those sentences to properly interpret the direction of the change. The results and statistics reported are correct, the changes solely refer to the discussion of the results (these changes do not change the general scientific conclusions of the article in any way).

A correction has been made to the Abstract (denoting that the pond originated larvae had low plasticity):

Where it says: “When transferred to streams, pond-originated larvae showed a **high** degree of plasticity, resembling the *morphology and* gene expression of stream-originated larvae *(reversion)*; *however* the same was not found for stream-originated larvae when transferred to ponds, where the expression of genes related to reduction-oxidation processes was increased, possibly to cope with environmental stress” 

It should say: “When transferred to streams, pond-originated larvae showed a **low** degree of plasticity, resembling the gene expression of non-transferred pond-originated larvae; the same was not found for stream-originated larvae when transferred to ponds, where the expression of genes related to reduction-oxidation processes was increased, possibly to cope with environmental stress.”

A correction has been made to the Discussion, first paragraph (denoting that the ancestral population is more plastic, not the derived):

Where it says: “Moreover, this study provides novel data on the ability of individuals to **adapt to the ancestral** habitat type *(regression)*, and the challenges of colonizing new habitats.”

It should say: “Moreover, this study provides novel data on the ability of individuals to **cope with new** habitat types, and the challenges of colonizing new habitats.”

A correction has been made to the Discussion, fifth paragraph (correcting the treatment name and adjusting the interpretation of the results):

Where it says: “We find that **P-S** individuals presented a gene expression profile that is intermediate between those of **pond** (origin) and **stream** (destination) larvae, showing some acclimatization to the **stream** environment *(regression)*. This is further supported by the morphological data (Figure 4 and Figure 5)—*the gill length of P-S individuals matched that of the pond individuals—yet the body condition index and the growth rate matched that of stream individuals (Figure 5); this is likely due to the abundance of food in this environment [47]. Conversely, S-P individuals presented a gene expression profile similar to individuals from streams (origin) but not to those from ponds (destination). The reduced acclimatization to the destination environment can also be seen in the morphological data, in which the body condition index and the gill length of S-P individuals is the same as that of S-S individuals (Figure 5). The only aspect of S-P individuals which did not match S-S individuals was the growth rate; this was higher than both non-transferred groups (P-P and S-S; Figure 4C and Figure 5). This* could be related to the fact that *streams* have lower oxygen content and food availability [38,39,47]. These differences are potentially interpreted as environmental stress and induce hormonal changes [65,66,67] and, thus lead to more rapid metamorphosis [68,69]. This is further supported by the fact that S-P individuals had enriched GO terms related to skeletal system development and mitotic chromosome condensation, which are functions associated with growth and metamorphosis.”

It should say: “We find that **S-P** individuals presented a gene expression profile that is intermediate between those of **stream** (origin) and **pond** (destination) larvae, showing some acclimatization to the **pond** environment. This is further supported by the morphological data (Figure 4 and Figure 5), which could be related to the fact that **ponds** have lower oxygen content and food availability [38,39,47]. These differences are potentially interpreted as environmental stress and induce hormonal changes [65,66,67] and, thus lead to more rapid metamorphosis [68,69]. This is further supported by the fact that S-P individuals had enriched GO terms related to skeletal system development and mitotic chromosome condensation, which are functions associated with growth and metamorphosis. Conversely, P-S individuals presented a gene expression profile similar to individuals from ponds (origin) but not to those from streams (destination). with the increase in body condition, likely being related with the abundance of food in this environment [47].”

A correction has been made to the Discussion, sixth paragraph (denoting that the ancestral population is more plastic, not the derived):

Where it says: “This explains why **pond**-originated salamanders were able to acclimatize to **streams** (regression), while the **stream**-originated larvae—*never having been in contact with the pond environment before*—could not acclimatize as easily to the conditions imposed by **ponds**.”

It should say: “This explains why **stream**-originated salamanders were able to acclimatize to **ponds**, while the **pond**-originated larvae—having incorporated some changes in order to cope with a more stressful environment—could not acclimatize as easily to the conditions imposed by **streams**.”

The authors apologize for this error and state that this does not change the general scientific conclusions of the article in any way (the variability in the study population is still partially due to plasticity and partially due to genetic adaptation, however the direction is reversed).

**Error in Figure**

In the original article, there was a mistake in Figure 4 as published. There was an extra significance symbol in the Gill size graph between the PP and PS treatment groups. The corrected Figure 4 appears below. The authors apologize for this error and state that this does not change the scientific conclusions of the article in any way.

**Figure 4:**
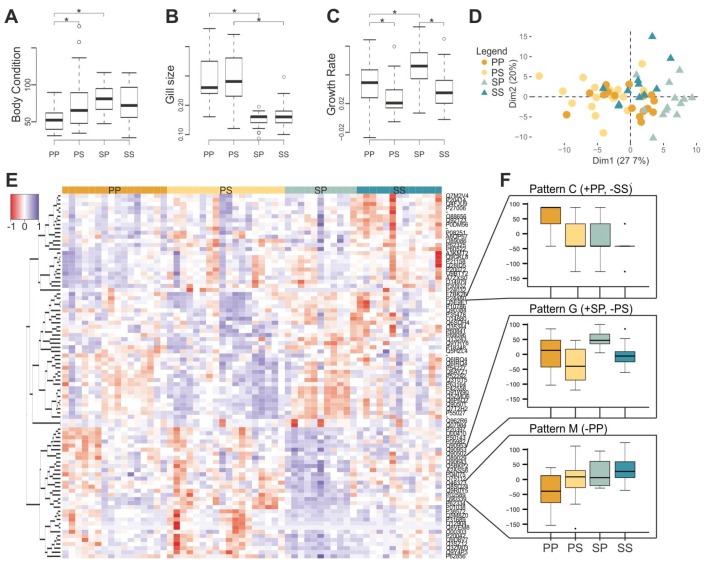
Transfer experiment results. Histograms representing (**A**) body condition index, (**B**) gill size, and (**C**) growth rate of pond individuals (PP), pond individuals transferred to streams (PS), stream individuals transferred to ponds (SP) and stream individuals (SS). (**D**) A posteriori principal component analysis of gene expression data depicting pond-originated (circles), stream-originated individuals (triangles), transferred (lighter), and non-transferred (darker). (**E**) Heat map of the differently expressed transcripts (89) between PP, PS, SP, and SS. (**F**) Expression patterns identified by self-organizing maps analysis based on differently expressed probes (+: Overexpression, −: Underexpression).

