# Supplementary material for: The Role of Plasticity and Adaptation in the Incipient Speciation of a Fire Salamander Population
Source: Genes (Basel). 2019 Oct 31;10(11):875. doi: 10.3390/genes10110875 (PMC6896149; doi:10.3390/genes10110875)
Supplement: Supplementary file 1 [file genes-10-00875-s001.zip › SabinoPinto_etal_SupMaterials_revised.docx]

SUPPLEMENTARY MATERIALS

TO

**The role of plasticity and adaptation on the incipient speciation of a fire salamander population**

Joana Sabino-Pinto^1,*,†^, Daniel J. Goedbloed^1,†^, Eugenia Sanchez^1,2^, Till Czypionka^3^, Arne W. Nolte^4^, Sebastian Steinfartz^5,*^

^1^ Department of Evolutionary Biology, Zoological Institute, Technische Universität Braunschweig, 38106 Braunschweig, Germany

^2^ Department of Biology, Stanford University, Stanford, CA 94305, USA

^3^ Laboratory of Aquatic Ecology and Evolutionary Biology, KU Leuven, 3000 Leuven, Belgium^4^ Department of Ecological Genomics, Institute for Biology and Environmental Sciences, University of Oldenburg, 26129 Oldenburg, Germany

^5^ University of Leipzig, Institute of Biology, Molecular Evolution and Systematics of Animals, 04103 Leipzig, Germany

* Corresponding authors: Joana Sabino-Pinto ([joanasabinopinto@gmail.com](mailto:joanasabinopinto@gmail.com)) and Sebastian Steinfartz: (steinfartz@uni-leipzig.de)

^†^Shared first co-authorship

**Supplementary Results**

*Translocation effect*

Individuals kept in their collection site were similar to the ones transferred to the other site of the same habitat type (e.g. PondA-PondA vs. PondA-PondB; Sup. Fig. 4). There were no differences related to gene expression.

Out of the 12,744 probes present in the microarray, 12,478 and 12,441 did not have missing values for the pond and the stream habitat datasets, respectively. *A priori* hierarchical clustering and PCAs revealed a lack of grouping of the samples according to translocation and site of origin for both habitats (Pond: F_1,30_ = 1.04, p-value = 0.385; Stream: F_1,20_ = 2.04, p-value = 0.064) (Sup. Fig. 4).

*Container effect*

Individuals kept inside the containers were similar to the ones captured outside (Sup. Fig. 5). There were no differences related to gene expression.

Out of the 12,744 probes present in the microarray, 12,415 did not have missing values. An *a priori* hierarchical clustering and PCA revealed a lack of grouping of the samples according to cage (inside vs. outside) (F_1,25_ = 0.05, p-value = 0.193) (Sup. Fig. 5).

**Supplementary Figures**


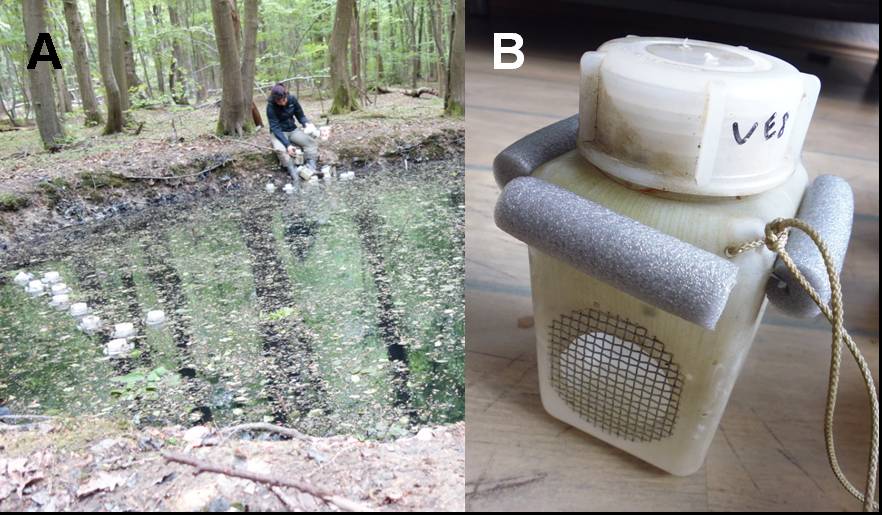


**Supplementary Figure 1.** Field setup. **A)** Placement of two sets of containers at KoV2. **B)** Example container used to house the larvae during the experiment.


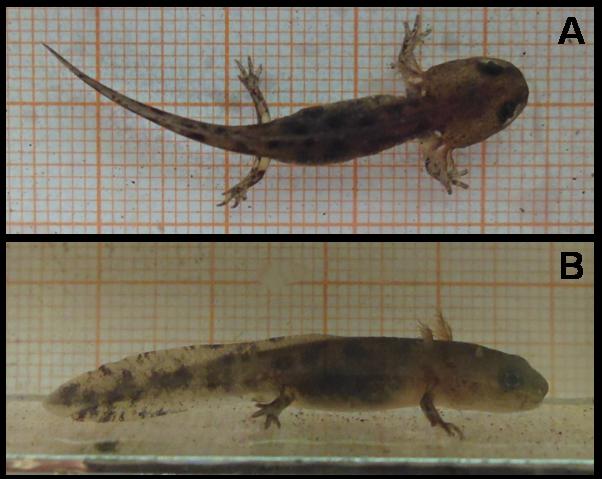


**Supplementary Figure 2.** Examples of top (A) and side (B) photos of the same salamander larva.


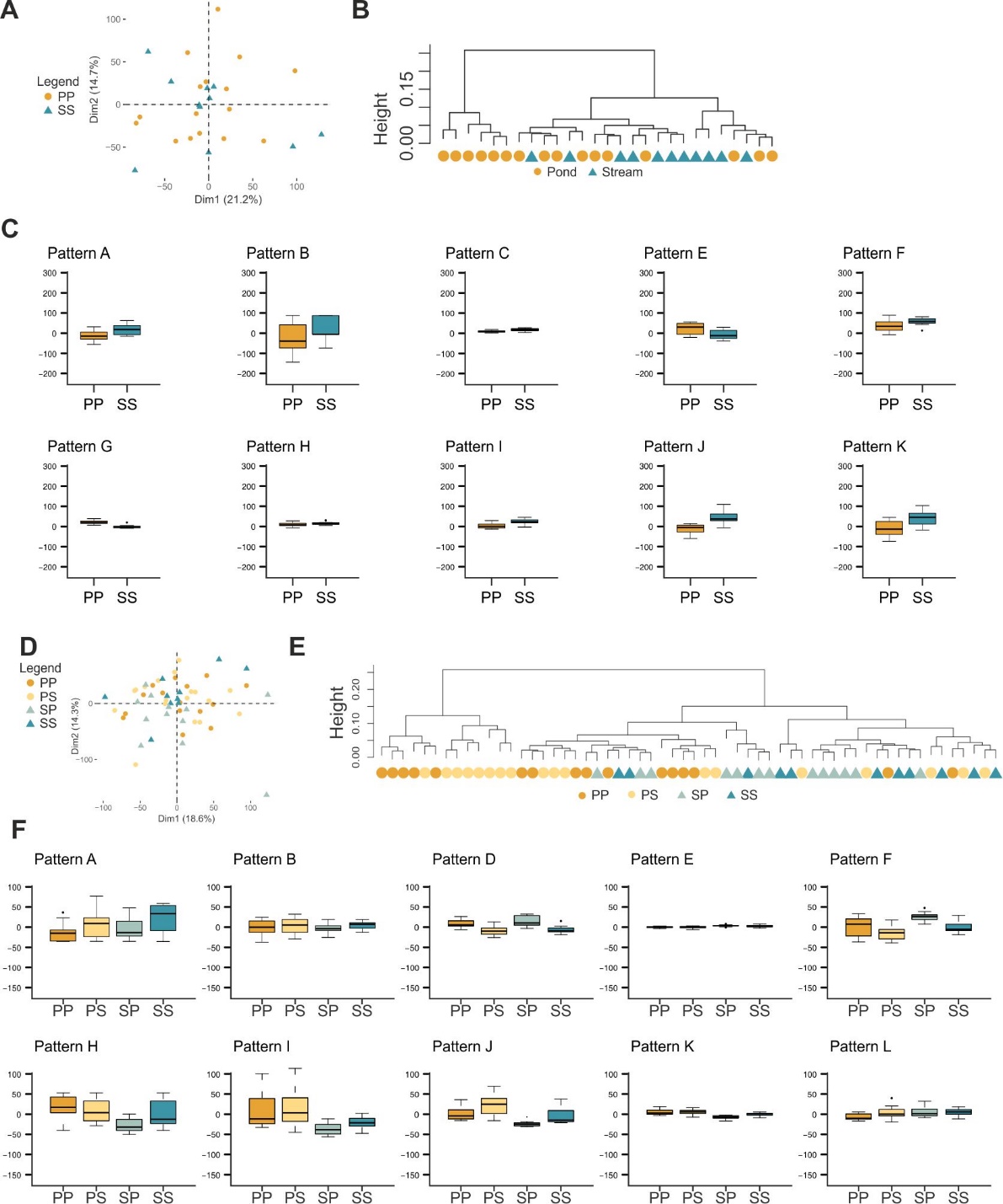


**Supplementary Figure 3.** Habitat type (A-C) and transfer (D-E) experiment results. **A)** and **D)** *a priori* principal component analysis, **B)** and **E)** hierarchical clustering based on a Pearson correlation distance matrix of gene expression data. **C)** and **F)** expression patterns identified by SOM analysis based on differently expressed probes without differences between groups. PP: pond individuals; PS: pond individuals transferred to streams; SP: stream individuals transferred to ponds; SS: stream individuals.


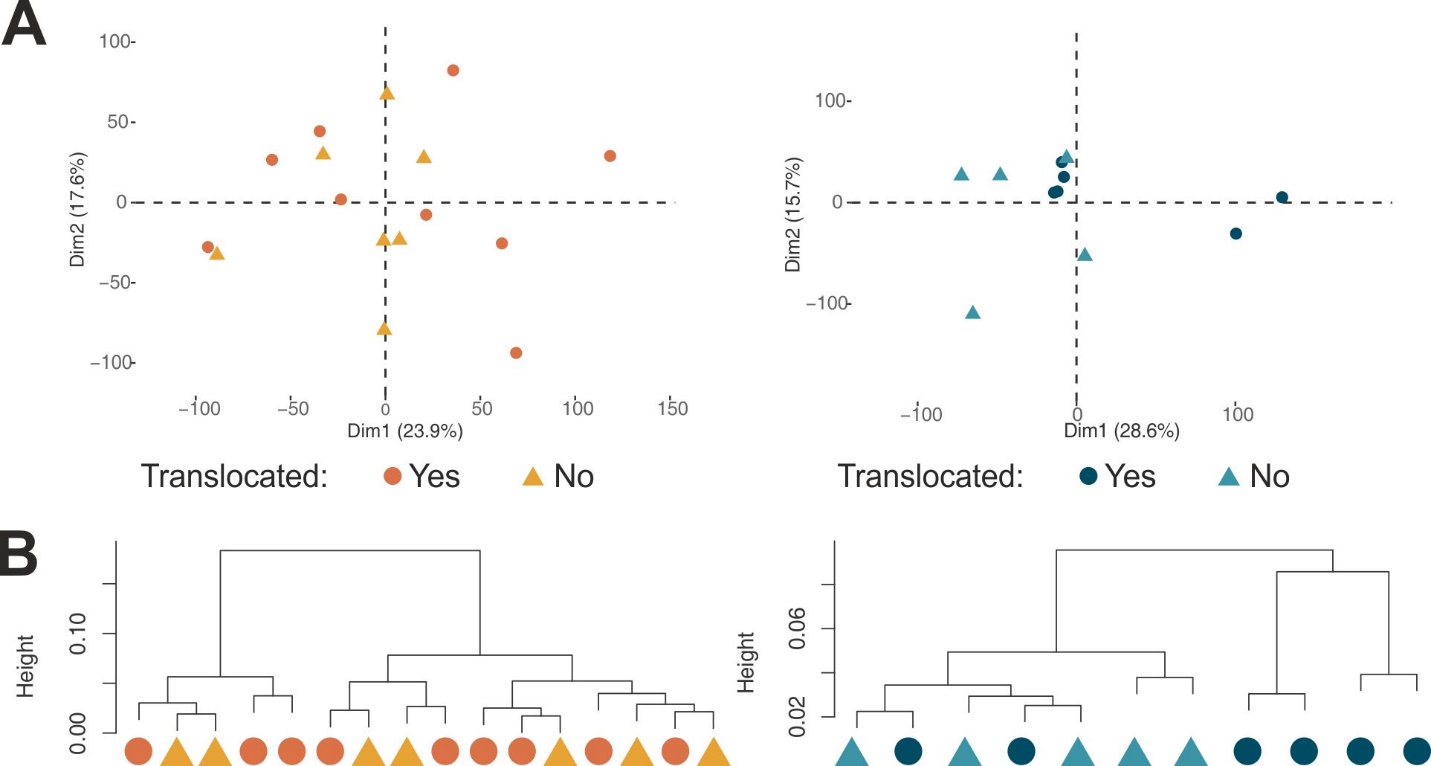


**Supplementary Figure 4.** Translocation effect experiment results. **A)** *A priori* principal component analysis, and **B)** hierarchical clustering based on a Pearson correlation distance matrix of gene expression data depicting non-translocated individuals (i.e. kept in the same site; triangles) and translocated ones (i.e. moved to the other site of the same habitat type; circles). Left side of A and B for pond individuals, right side of A and B for stream individuals.


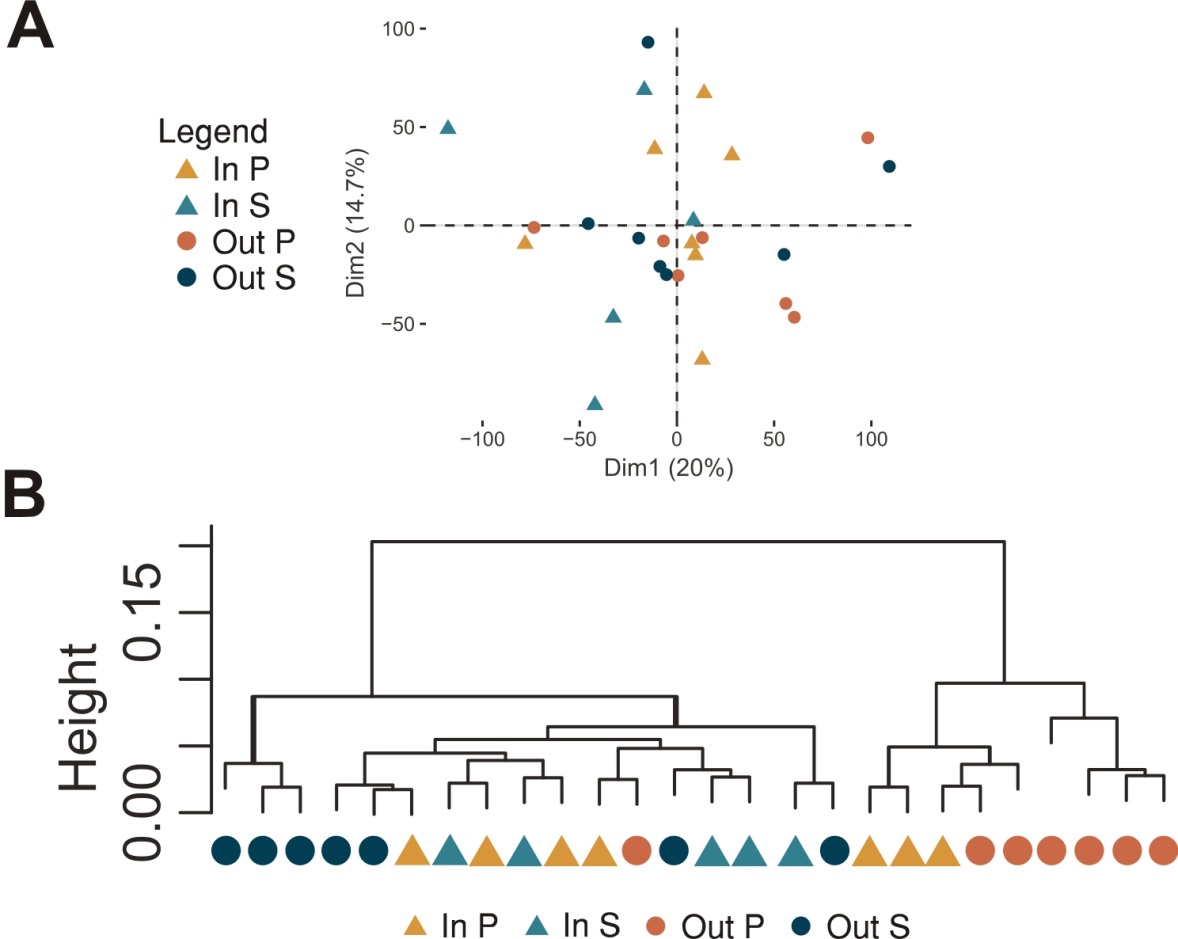


**Supplementary Figure 5.** Cage effect experiment results. **A)** *A priori* principal component analysis, and **B)** hierarchical clustering based on a Pearson correlation distance matrix of gene expression data depicting experimental individuals (triangles) and free swimming ones (circles). P for pond individuals, S for stream individuals.

# Supplementary Tables

**Supplementary Table 1.** Sample sizes. Groups are coded in a from-to fashion based on site ID (B=KoGb, C=KoGc, E=KoE and V=KoV2).

**Supplementary Table 2.** List of probes differently expressed between larvae originated from ponds and streams in the Kottenforst. “LDA effect size” and “p-value” according to LEfSe, “Functions” according to QuickGO, transcript description according to Annocript.

**Supplementary Table 3.** GO term (code and name) enrichment for habitats. Site: habitat on which terms were upregulated. Significant: p(FDR): Significance levels of GO terms that were enriched with pFDR < 0.05

**Supplementary Table 4.** List of probes differently expressed between larvae originated from ponds and streams in the Kottenforst according to Czypionka et al. (2015), Goedbloed et al. (2016) and this study. “Function” according to QuickGO, transcript description according to Annocript.

**Supplementary Table 5.** List of probes differently expressed between larvae from the Kottenforst originated from ponds and kept in ponds (PP), originated from ponds and transferred to streams (PS), originated from streams and transferred to ponds (SP), and originated from streams and kept in streams (SS). “LDA effect size” and “p-value” according to LEfSe, “Functions” according to QuickGO, transcript description according to Annocript.

**Supplementary Table 6.** GO term (code and name) enrichment for treatments. Treatment: treatment on which terms were upregulated. Significant: p(FDR): Significance levels of GO terms that were enriched with pFDR < 0.05

**Supplementary Table 7.** Morphological raw data. (B=KoGb, C=KoGc, E=KoE and V=KoV2).
